# A large expert-annotated single-cell peripheral blood dataset for hematological disease diagnostics

**DOI:** 10.1038/s41597-025-06223-x

**Published:** 2025-11-11

**Authors:** Sayedali Shetab Boushehri, Salome Kazeminia, Armin Gruber, Christian Matek, Karsten Spiekermann, Christian Pohlkamp, Torsten Haferlach, Carsten Marr

**Affiliations:** 1 Computational Health Center, Helmholtz Munich – German Research Center for Environmental Health, Neuherberg, Germany; 2 Data & Analytics, Pharmaceutical Research and Early Development (pRED), Roche Innovation Center Munich (RICM), Penzberg, Germany; 3 TUM School of Computation, Information and Technology, Technical University of Munich, Munich, Germany; 4 Department of Medicine III, University Hospital, LMU Munich, Munich, Germany; 5 German Cancer Consortium (DKTK), Heidelberg, Germany; 6 German Cancer Research Center (DKFZ), Heidelberg, Germany; 7 Munich Leukemia Laboratory, Munich, Germany; 8 Munich Center for Machine Learning (MCML), Munich, Germany

**Keywords:** Leukaemia, Cellular imaging

## Abstract

Distinguishing cell types in a peripheral blood smear is critical for diagnosing blood diseases, such as leukemia subtypes. Artificial intelligence can assist in automating cell classification. For training robust machine learning algorithms, however, large and well-annotated single-cell datasets are pivotal. Here, we introduce a large, publicly available, annotated peripheral blood dataset comprising >40,000 single-cell images classified into 18 classes by cytomorphology experts from the Munich Leukemia Laboratory, the largest European laboratory for blood disease diagnostics. By making our dataset publicly available, we provide a valuable resource for medical and machine learning researchers and support the development of reliable and clinically relevant diagnostic tools for diagnosing hematological diseases.

## Background & Summary

Microscopic examination and classification of blood cells play a crucial role in diagnosing hematological diseases. This process involves evaluating the morphology of leukocytes and is typically performed by human experts trained over years. Like other diagnostic tasks, it is repetitive, time-consuming, and susceptible to intra- and inter-observer variation^1^. One promising solution is the development of automatic single-cell classifiers using machine learning, which can substantially reduce the time and effort required by experts^2^. Deep learning, in particular, has been used for diagnosing hematological diseases from single-cell images in peripheral blood^3–9^ and bone marrow^10–12^.

As supervised deep learning crucially relies on large amounts of annotated data, a current lack of large datasets creates a bottleneck for improving the accuracy of classifiers^13^. This work presents the largest publicly available, expert-annotated dataset of peripheral blood single-cells, with over 40,000 images. While our dataset is being published here for the first time, it has been used in previous studies^4,5,14–17^.

## Methods

### Ethics declaration

Informed consent was obtained indirectly at the time of routine collection for possible research. All patients in the MLL23 dataset were at least 18 years old. Ethics approval was granted by the Ethics Committee of LMU Munich (reference number 25-0744).

**Fig. 1:**
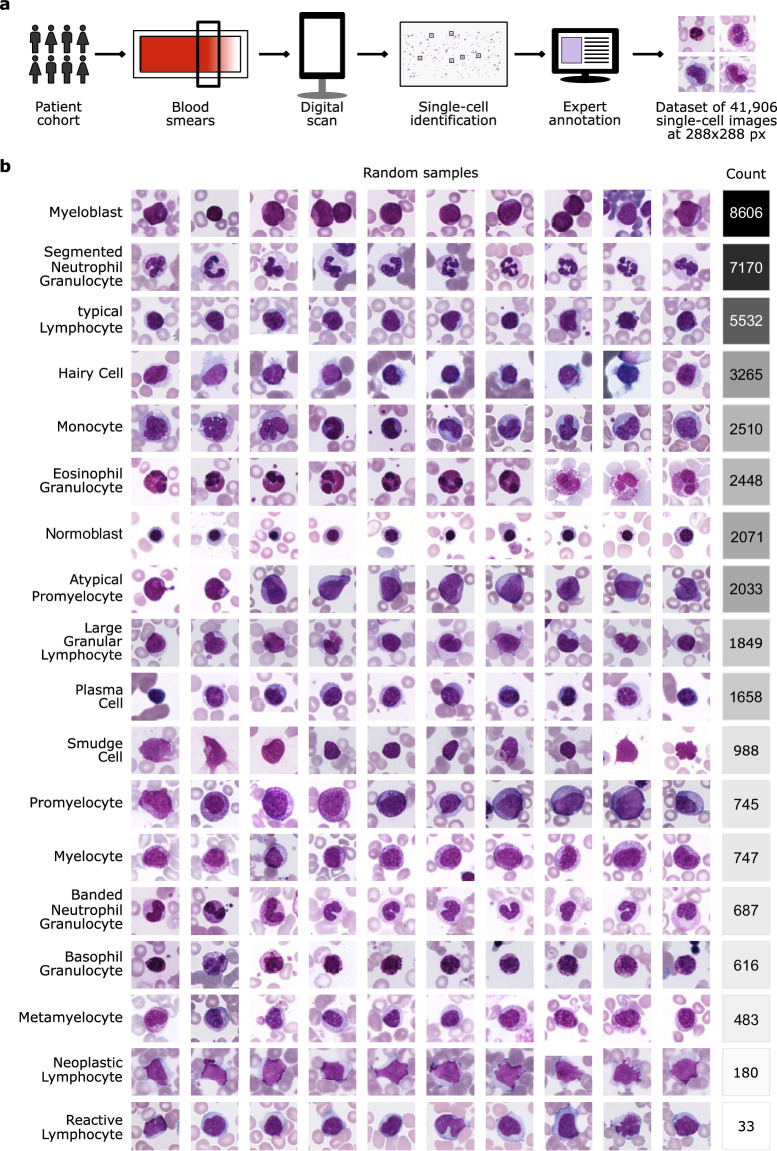
A fully annotated single-cell peripheral blood dataset. (**a**) Workflow of generating the imaging dataset at the Munich Leukemia Laboratory. (**b**) The MLL23 dataset contains 18 classes with varying numbers of images per class. Ten representative images per class are depicted to provide an overview of the dataset.

The data acquisition process at the Munich Leukemia Laboratory comprised several steps (see also Hehr *et al*.^4^). Blood samples and smears were collected between 2021 and 2024 from patients with a wide distribution of hematological diagnoses. A patient cohort with blood samples from adult patients who gave informed consent to the use of their data for research purposes was selected. Blood smears were stained using the Pappenheim method and scanned using a fully automated scanning device (Metafer software platform, MetaSystems, Altlussheim, Germany), which was modified in its technical settings for this application. Image acquisition was performed using an automatic autofocus system integrated in the scanning device, without manual focus adjustments. Slides were first scanned with a 10x objective to obtain an overview image. Cell detection was performed using the Metasystems Metafer software. After applying a segmentation threshold and a logarithmic color transformation, stained cells with an object size between 40–800 μm^2^ were detected and stored in a gallery. Each gallery image was assigned to a quality level using a classifier to determine cell density and immediate cell neighborhood. High-quality cells identified in the 10x overview images were then re-scanned using a 40x objective. The resulting 41,906 images of single nucleated cells comprise 288 × 288 pixels and 25 μm × 25 μm, corresponding to a resolution of 11.52 pixels per μm. Note that the occasional white bars at the edges of some images result from edge effects when cells are located near boundaries of the scanned field of view. To maintain uniformly sized square images, we padded images with white pixels, matching the background, regardless of horizontal or vertical orientation. Subsequently, five human expert examiners at the Munich Leukemia Laboratory annotated the images, assigning each single cell to one out of 18 classes (Fig. 1a).

We reduced the dataset to 41,621 cells by deleting duplicate images. Some duplicate images also had differing labels, corresponding to indecisive borderline cases. Note that some cells are depicted in two or more images, but with differing focus or cropping. Also, dysplastic cells were excluded from the dataset to ensure clarity in cell type classification.

In the group of lymphoid cells, there are mature ‘typical lymphocytes’ (number of single-cell images = 5,532) and ‘atypical lymphocytes’ like plasma cells (1,658), ‘large granular lymphocytes’ (1,849), ‘reactive lymphocytes’ (33), ‘hairy cells’ (3,265) and other ‘neoplastic lymphocytes’ (180), as well as ‘smudge cells’ (988). In comparison, the group of myeloid cells is divided into mature cells like band ‘neutrophil granulocytes’ (687), ‘segmented neutrophil granulocytes’ (7,170), ‘eosinophil granulocytes’ (2,448), ‘basophil granulocytes’ (616), ‘monocytes’ (2510), and immature cells like ‘myeloblasts’ (8,606), ‘metamyelocytes’ (483), ‘promyelocytes’ (745), ‘myelocytes’ (747), and ‘atypical promyelocytes’ (2,033). Lastly, ‘normoblasts’ (2071) are also present in the dataset. The cell types occur with specific frequencies in the peripheral blood in healthy and pathological patients. Due to the Munich Leukemia Laboratory’s focus on hematologic neoplasms, the dataset is inherently imbalanced in terms of the number of images per class. For instance, it contains over 8,000 myeloblasts but only 33 reactive lymphocytes (Fig. 1b).

## Technical Validation

All data in the MLL23 dataset originate from routine diagnostics at the Munich Leukemia Laboratory (MLL), one of Europe’s largest reference centers for hematologic malignancies. As part of the standard diagnostic workflow, all cytological preparation and image acquisition is subject to stringent internal quality control and external benchmarking, including regular participation in inter-laboratory ring trials and accreditation processes. Each image was labeled by one of five expert examiners at MLL, assigning single cells to one of 18 morphologically defined classes.

A limitation of the MLL23 dataset is the natural rarity of certain cell types in peripheral blood samples. Because these minority cell types occur infrequently under both normal and pathological conditions, we cannot increase their representation during data collection. This biological constraint directly results in class imbalance, which reflects real-world distributions but poses challenges for training machine learning models on this dataset.

## Data Availability

The dataset is available at 10.5281/zenodo.14277609. It comprises 18 ZIP files, each named after a specific cell type (e.g., basophil.zip). Each ZIP file contains high-quality TIFF images of individual cells belonging to the corresponding class, with file names following a consistent format that includes the class name and a unique identifier (e.g., basophil_0001.TIF).

## Code availability

No custom code was used in this study. All analyses were performed without the need for proprietary or bespoke software.
